# Modeling the Footprint and Equivalent Radiance Transfer Path Length for Tower-Based Hemispherical Observations of Chlorophyll Fluorescence

**DOI:** 10.3390/s17051131

**Published:** 2017-05-16

**Authors:** Xinjie Liu, Liangyun Liu, Jiaochan Hu, Shanshan Du

**Affiliations:** 1 Key Laboratory of Digital Earth Science, Institute of Remote Sensing and Digital Earth, Chinese Academy of Sciences, Beijing 100094, China; liuxj@radi.ac.cn (X.L.); hujc@radi.ac.cn (J.H.); duss@radi.ac.cn (S.D.); 2 University of Chinese Academy of Sciences, Beijing 100049, China

**Keywords:** tower-based sensing, solar-induced chlorophyll fluorescence, radiance transfer path length, bi-hemispherical spectral observation, footprint

## Abstract

The measurement of solar-induced chlorophyll fluorescence (SIF) is a new tool for estimating gross primary production (GPP). Continuous tower-based spectral observations together with flux measurements are an efficient way of linking the SIF to the GPP. Compared to conical observations, hemispherical observations made with cosine-corrected foreoptic have a much larger field of view and can better match the footprint of the tower-based flux measurements. However, estimating the equivalent radiation transfer path length (ERTPL) for hemispherical observations is more complex than for conical observations and this is a key problem that needs to be addressed before accurate retrieval of SIF can be made. In this paper, we first modeled the footprint of hemispherical spectral measurements and found that, under convective conditions with light winds, 90% of the total radiation came from an FOV of width 72°, which in turn covered 75.68% of the source area of the flux measurements. In contrast, conical spectral observations covered only 1.93% of the flux footprint. Secondly, using theoretical considerations, we modeled the ERTPL of the hemispherical spectral observations made with cosine-corrected foreoptic and found that the ERTPL was approximately equal to twice the sensor height above the canopy. Finally, the modeled ERTPL was evaluated using a simulated dataset. The ERTPL calculated using the simulated data was about 1.89 times the sensor’s height above the target surface, which was quite close to the results for the modeled ERTPL. Furthermore, the SIF retrieved from atmospherically corrected spectra using the modeled ERTPL fitted well with the reference values, giving a relative root mean square error of 18.22%. These results show that the modeled ERTPL was reasonable and that this method is applicable to tower-based hemispherical observations of SIF.

## 1. Introduction

Accurate estimation of gross primary production (GPP) is of great importance to global change research and ecosystem monitoring. In recent years, the solar-induced chlorophyll fluorescence (SIF) retrieved from hyperspectral data has become a new powerful tool for the estimation of the ecosystem GPP [1,2,3,4,5,6].

The eddy covariance (EC) technique has dramatically enhanced our understanding of inter- and intra-annual variations in carbon fluxes at the ecosystem scale [7]. However, EC measurements are dispersed and only cover quite limited regions [8]. For continuous global monitoring of carbon exchange, satellite remote sensing is needed. However, the data from EC observations and satellite remote sensing cannot be linked directly due to the mismatch of spatial scales [9,10]. To deal with this problem, continuous tower-based spectral observations in coordination with EC measurements is an efficient solution and can serve as a bridge between the eddy covariance (EC) measurements and satellite-based remote sensing data [9,10,11,12].

In the last decade, a growing number of automatic tower-based spectroscopy systems have been initiated with the aim of making long-term, unattended observations of vegetation using different instruments and configurations. Balzarolo et al. [11] provided a review of the current optical systems and methods used for continuous vegetation optical sampling across European EC sites. Several hyperspectral systems have been proposed for continuous sun-induced fluorescence (SIF) detection out of the automatic optical systems currently installed at the European EC sites. These include the Multiplexer Radiometer Irradiometer (MRI) [12], the HyperSpectral Irradiometer (HSI) [13], the TriFLEX [14], the UNIEDI System [15], the FluoSpec [16], and the SIF-Sys [17]. For passive SIF observations, the spectral resolution requirements are generally sub-nanometer [18]. Therefore, most of these systems provide high spectral resolution (FWHM ≤ 1 nm) and their spectral ranges cover the SIF emission region (650–850 nm) [19]. Within this framework, both SpecNet [9] and the European equivalent COST Action ES0903 (EUROSPEC, http://cost-es0903.fem-environment.eu/) aim to establish common ground-based instruments, define measurement protocols for tower-based spectral observations and to promote the development of reliable, scale-appropriate and cost-effective optical global flux tower measurements. 

However, the mismatch between the footprints of different spectral flux observations is a key problem [10,13]. Since the goal of the tower-based observations is to provide spectral signals in coordination with the flux data, the footprints of the two systems should match. A number of factors contribute to the flux footprint variability, including wind direction and speed, measurement height, and vegetation structure [20,21]. A series of empirical flux footprint models (e.g., [22,23,24,25]) have been proposed to estimate the footprint of flux measurements made under different conditions. 

**Figure 1:**
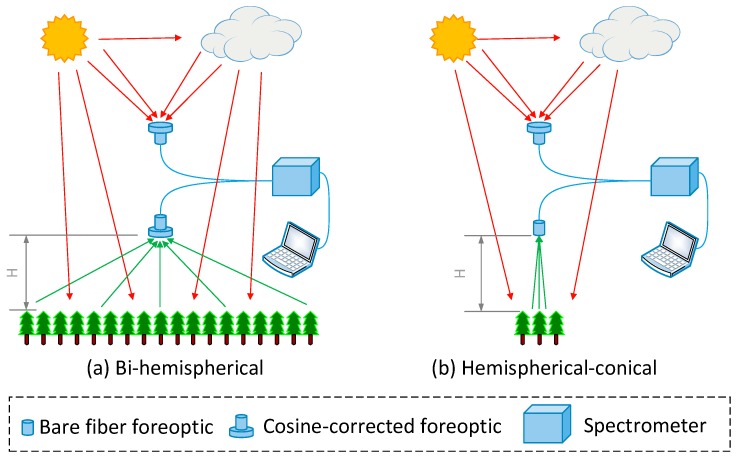
Illustration of the bi-hemispherical and hemispherical-conical configurations of in situ spectral measurements.

The footprint of a spectral measurement is determined by the instrument configuration and a configuration suitable for the specific purpose of the measurements should be selected. Tower-based spectral measurements involve the measurement of the down-welling (incoming) and up-welling (both reflected and emitted) radiation fluxes from the canopy. Three main instrument configurations have been applied to in situ optical observations to quantify incoming and reflected radiation: bi-conical, hemispherical-conical and bi-hemispherical [10,26]. The bi-hemispherical and hemispherical-conical configurations are the most commonly used for tower-based and long-term spectral observation [11]. The setups of the bi-hemispherical and hemispherical-conical configurations are shown in Figure 1. Both configurations use cosine-corrected foreoptic pointed at the zenith to acquire the downwelling irradiance values. For the up-welling measurements, hemispherical-conical configurations use a conical fore-optic (bare fiber) at nadir or off-nadir with a small field of view (FOV) (such as 25°) whereas bi-hemispherical configurations use a cosine-corrected foreoptic view at nadir with a large FOV of 180°. Obviously, compared to the conical observations, the hemispherical observations of the up-welling radiation have the advantage of sampling a wider area [10] and hence provide a footprint that is more similar to that of the EC measurements.

However, for bi-hemispherical measurements with a cosine-corrected foreoptic, the radiation transfer path turns out to be different from that for conical observations. The radiation transfer path length (RTPL) increases with the view zenith angle in the form of a secant function. For conical observations made with a bare fiber, as the FOV is very small (perhaps 25°), the equivalent radiation transfer path length (ERTPL) is approximately equal to the height of the sensor above the surface. However, for hemispherical measurements, the FOV is 180° and so the ERTPL will be much larger than the height of the sensor. For ground-based SIF retrieval, the most commonly used bands are the telluric oxygen absorption bands, which are very sensitive to the atmospheric radiation transfer. The absorption depth of the oxygen absorption bands is mainly related to the RTPL. According to the study by Liu et al. [27], a bias of 10 m in the sensor’s height will lead to a bias of about 0.1 mW/m^2^/nm/sr in the retrieved SIF at the O_2_-A band, which is not negligible for SIF retrieval. Therefore, accurate atmospheric correction (in other words, accurate estimation of the ERTPL) is of vital importance for tower-based observations of SIF, even if the height of the sensor above the canopy is only some tens of meters. Consequently, the ERTPL of bi-hemispherical measurements must be analyzed. However, according to our knowledge, no such analysis has been reported until now.

Therefore, the modeling of the footprint and ERTPL of bi-hemispherical spectral measurements is important in the context of tower-based measurements of SIF. The aims of this paper are: (1) to analyze the footprints of tower-based hemispherical spectral observations, and also the match between these footprints and those of tower-based flux observations; (2) to determine the ERTPL of hemispherical spectral observations and; (3) to evaluate the derived ERTPL using simulations and to test the validity of the atmospheric correction by using the derived ERTPL for tower-based SIF retrieval.

## 2. Materials and Methods

### 2.1. Data Simulation

In order to model the ERTPL, we built a simulated dataset by integrating the Soil Canopy Observation, Photochemistry and Energy fluxes v1.7 (SCOPE v1.7) [28] and the MODerate resolution atmospheric TRANsmission 5 (MODTRAN 5) [29] models. The SCOPE model was used for the simulation of the canopy reflectance and SIF spectra, while the MODTRAN 5 model was used for the simulation of the atmospheric radiation transfer parameters. Four different levels of chlorophyll content and leaf area index (LAI), and five different typical leaf inclination distribution conditions were used in the SCOPE model to represent different (80 different simulated samples in total) vegetation conditions. For the O_2_-A band we studied in this paper, the absorption by oxygen is the dominant characteristics of atmospheric radiation transfer, and is mainly influenced by the observation geometry. So the atmospheric condition (including aerosol model, aerosol optical depth, water vapor and ozone column, etc.) were set as fixed values. The main parameters used in the SCOPE and MODTRAN 5 models are listed in Table 1. All other parameters were set to their default values. The spectral resolution (full width at half maximum) of the simulated spectra was 0.3 nm and the spectral sampling interval was 0.15 nm.

Assuming the surface is Lambertian, with the simulation results from SCOPE and MODTRAN 5, the upwelling radiance at the height of the observation platform can be calculated as [30]:

(1) $$L_{H}\left(\theta ,\varphi \right)=\frac{L_{0}\left(\theta ,\varphi \right)\cdot T_{↑}\left(\theta \right)}{1-\rho \left(\theta ,\varphi \right)S}+L_{path}\left(\theta ,\varphi \right)$$

where $L_{H}$ is the upwelling radiance arriving at the observation platform at a height *H* above the target surface; $L_{0}$ is the radiance at the top of the canopy, which contains a contribution from both reflected radiance and emitted SIF; $T_{↑}$ is the upwelling transmittance of the atmosphere; ρ is the surface reflectance; S is the atmospheric spherical albedo accounting for multiple scattering between atmosphere and surface; and $L_{path}$ is the atmospheric path radiance. For tower-based observations, the platform height is relatively low. The atmospheric scattering effect was, therefore, neglected in this study and Equation (1) can be simplified as:

(2) $$L_{H}\left(\theta ,\varphi \right)=L_{0}\left(\theta ,\varphi \right)\cdot T_{↑}\left(\theta \right)$$

Using Equation (2), $L_{H}$ for different values of the VZA and RAA can be calculated using the simulations made by SCOPE and MODTRAN 5. 

### 2.2. Footprint of Spectral Observation

The measured upwelling irradiance (E) can be calculated by integrating the radiance (L) over all the propagation directions (defined using the view zenith angle (θ) and view azimuth angle (φ)) within the FOV. For hemispherical measurements, neglecting the influence of atmospheric scattering, the measured irradiance of the signal within the FOV can be expressed as [13,31,32]:

(3) $$E\left(\mathrm{FOV}\right)=\int_{0}^{2\pi }\int_{0}^{\frac{FOV}{2}}L_{0}\left(\theta ,\varphi \right)\cdot T_{↑}\left(\theta \right)\cdot \cos\theta \cdot \sin\theta d\theta d\varphi .$$

where $T_{↑}\left(\theta \right)$ is the upwelling atmospheric transmittance at the given view zenith angle, θ. For bands free from atmospheric absorption, the transmittance can be assumed independent of the RTPL (i.e., $T_{↑}$ is independent of θ). Assuming that the ground surface is homogeneous and Lambertian (i.e., L is independent of the propagation direction), Equation (3) can be expressed as:

(4) $$E\left(\mathrm{FOV}\right)=2\pi \cdot L_{0}\cdot T_{↑}\cdot \int_{0}^{\frac{\mathrm{FOV}}{2}}\cos\theta \cdot \sin\theta d\theta =\pi \cdot L_{0}\cdot T_{↑}\cdot {\left(\sin\theta \right)}^{2}$$

**Figure 2:**
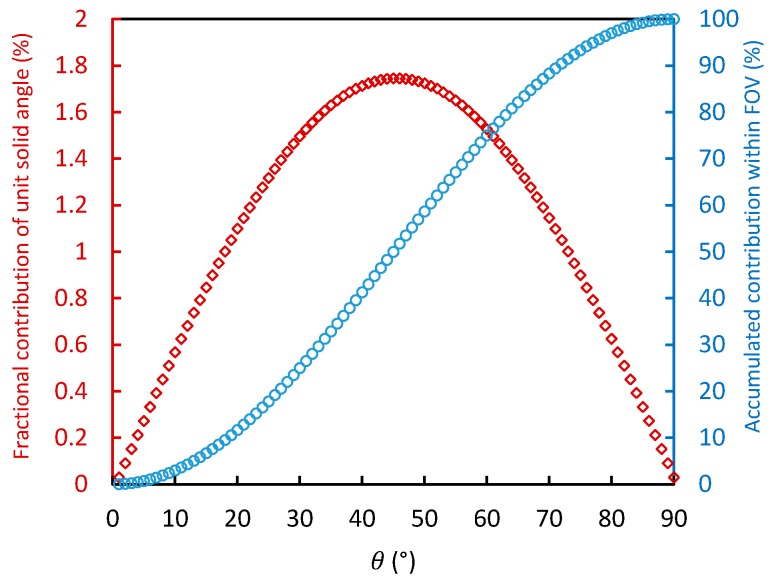
Fractional contribution of the signal coming from each 1° interval of the view zenith angle to the hemispherical irradiance (red diamonds) together with the accumulated contribution of the signal within the FOV (blue circles).

According to Equation (4), for hemispherical measurements made with the cosine-corrected foreoptic, the measured irradiance of the signal within the FOV is proportional to the square of sinθ. Using Equation (4) and discretizing the FOV to 1° intervals, it is possible to model the fractional contribution of the signal originating from each 1° interval of the zenith angle to the hemispherical irradiance (see the blue circles in Figure 2), as well as the accumulated contribution of the signal within the FOV (see the red diamonds in Figure 2). It can be seen from Figure 1 that the signal within an FOV of 72° contributes 90% of the total hemispherical irradiance, of which the maximum contribution comes from a view zenith angle of around 45°. 

### 2.3. ERTPL Modeling Based on Theoretical Derivation

The RTPL for hemispherical observations made with cosine-corrected foreoptic is more complex than for conical observations made using a bare fiber. When the FOV is as large as 180°, the ERTPL can no longer be estimated as being equal to the height of the sensor above the target surface. For spectral measurements made at the atmospheric windows, the influence of atmospheric radiation transfer on tower-based observations can be ignored as the height of the sensor above the target surface is usually only tens of meters. However, SIF observations are made at the atmospheric absorption bands and these are very sensitive to the RTPL. Therefore, for hemispherical observations, accurate modeling of the ERTPL is important.

According to Equation (3), the upwelling hemispherical irradiance observed with the cosine-corrected foreoptic can be expressed as:

(5) $$E_{cos}=\int_{0}^{2\pi }\int_{0}^{\frac{\pi }{2}}L_{0}\left(\theta ,\varphi \right)\cdot T_{↑}\left(\theta \right)\cdot \cos\theta \cdot \sin\theta d\theta d\varphi$$

Assuming that the surface is homogeneous and isotropic, and ignoring the bidirectional reflectance effect of the surface and the directional characteristics of SIF emission (i.e., $L_{0}$ is independent of θ and φ), the observed upwelling irradiance can be expressed as:

(6) $$E_{cos}=L_{0}\cdot \int_{0}^{2\pi }\int_{0}^{\frac{\pi }{2}}T_{↑}\left(\theta \right)\cdot cos\left(\theta \right)\cdot sin\left(\theta \right)d\theta d\varphi =2\pi L_{0}\cdot \int_{0}^{\frac{\pi }{2}}T_{↑}\left(\theta \right)\cdot cos\left(\theta \right)\cdot sin\left(\theta \right)d\theta$$

On the other hand, the observed upwelling irradiance can be expressed as the product of the upwelling irradiance at top of canopy ($E_{0}$) and the equivalent transmittance between the canopy and the sensor ($\bar{T_{↑}}$):

(7) $$E_{cos}=E_{0}\cdot \bar{T_{↑}}=\bar{T_{↑}}\cdot \int_{0}^{2\pi }\int_{0}^{\frac{\pi }{2}}L_{0}\left(\theta ,\varphi \right)\cdot \cos\theta \cdot \sin\theta d\theta d\varphi =\bar{T_{↑}}\cdot \pi L_{0}$$

Combining Equations (6) and (7), the equivalent transmittance can be calculated as:

(8) $$\bar{T_{↑}}=\frac{2\pi L_{0}\cdot \int_{0}^{\frac{\pi }{2}}T_{↑}\left(\theta \right)\cdot cos\left(\theta \right)\cdot sin\left(\theta \right)d\theta }{\pi L_{0}}=2\cdot \int_{0}^{\frac{\pi }{2}}T_{↑}\left(\theta \right)\cdot cos\left(\theta \right)\cdot sin\left(\theta \right)d\theta$$

**Figure 3:**
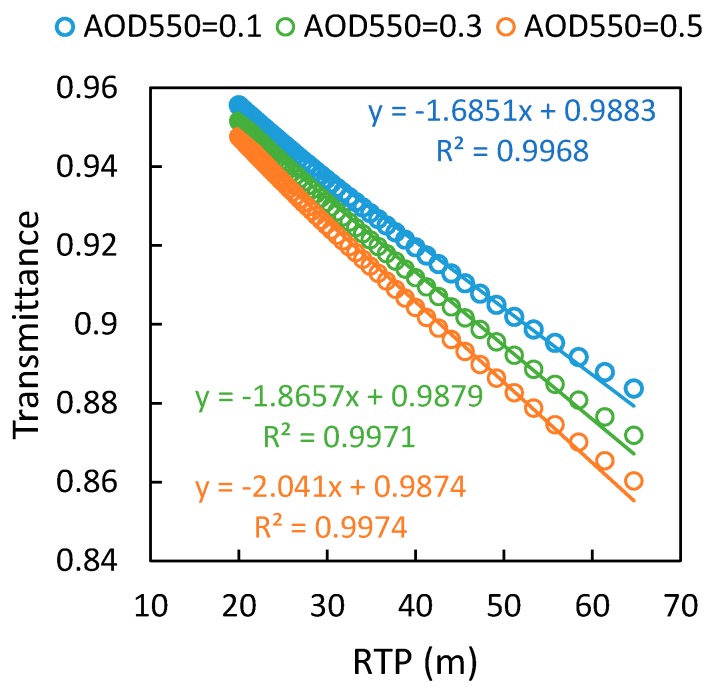
Variation of the transmittance of up-welling radiation at 761.1 nm with the RTPL according to simulations made by MODTRAN 5. The AOD_550_ is set as 0.1, 0.3, and 0.5; the sensor height is 20 m and the range of the view zenith angle is 0–72°; the other parameters are set as given in Table 1.

For the oxygen absorption bands, the transmittance is mainly related to the RTPL. Figure 3 shows the variation of the transmittance of the up-welling radiation with RTPL, based on simulations made by MODTRAN 5 with different aerosol optical depth (AOD_550_ is set as 0.1, 0.3, and 0.5). As there is no absorption by water and ozone at the wavelength of 761.1 nm we studied, the water vapor column is fixed as 3 g/cm^2^, and the ozone column is fixed as 300 DU. The view zenith angle is set to the range 0–72° with a sensor height of 20 m above the target surface, and the corresponding range of the RTPL is about 20–65 m. The results show that the transmittance is approximately linearly related to the RTPL within the range we tested (with R^2^ > 0.99). As shown in Figure 2, the signal within an FOV of 72° contributes more than 90% of the total hemispherical irradiance. So when estimating the equivalent transmittance for hemispherical observations, it is reasonable to assume a linear relation between $T_{↑}$ and RTPL when the atmospheric condition is fixed. Based on this assumption, $\bar{T_{↑}}$ and $T_{↑}\left(\theta \right)$ in Equation (8) can be replaced by the ERTPL and RTPL (θ):

(9) $$\mathrm{ERTPL}=2\cdot \int_{0}^{\frac{\pi }{2}}RTP\left(\theta \right)\cdot cos\left(\theta \right)\cdot sin\left(\theta \right)d\theta =2\cdot \int_{0}^{\frac{\pi }{2}}H\cdot sec\left(\theta \right)\cdot cos\left(\theta \right)\cdot sin\left(\theta \right)d\theta =2H$$

where H is the height of the sensor above the target surface.

According to this analysis, we can conclude that the ERTPL of hemispherical spectral observations with cosine-corrected foreoptic is about twice the height of the sensor above the target surface.

## 3. Results

### 3.1. Matching between the Footprints of Spectral and Flux Observations

To investigate the matching between the footprints of spectral and flux observations, the footprint of the flux measurements needs to be modeled. In contrast to spectral observations, estimating the footprint of flux measurements is more complex due to the influence of the atmosphere. 

Kljun et al. [25] presented a two-dimensional parameterisation for flux footprint prediction (FFP). Unlike other existing fast analytical footprint models, the FFP parameterisation is valid for a wide range of boundary layer stratifications and receptor heights, as well as for non-Gaussian turbulence. Therefore, in this study, FFP was employed for the simulation of the flux measurement footprint. The Xiao Tangshan EC site, located at the National Precision Agriculture Demonstration Base in the town of Xiao Tangshan, north of Beijing, China (40.17° N,116.39° E), was selected as a test site, and parameters measured on 18 April 2016 were used (details listed in Table 2). Using the online FFP (http://footprint.kljun.net/), a two-dimensional discrete footprint function at a height of 20 m for convective conditions was then modeled—shown as the red lines in Figure 3. 

According to Equation (4), it is possible to model the cumulative footprint contours of the hemispherical spectral observations made at nadir at a height of 20 m above the target surface. These are shown as the black lines in Figure 3. On the other hand, for the conical observations, the radiance measured by the bare fiber at nadir comes from a circular area on the ground with a radius of $H\cdot \tan\left(\frac{FOV}{2}\right)$, where H is the height of the sensor above the target surface. The source area for these measurements is marked as the blue circular area in Figure 3.

**Figure 4:**
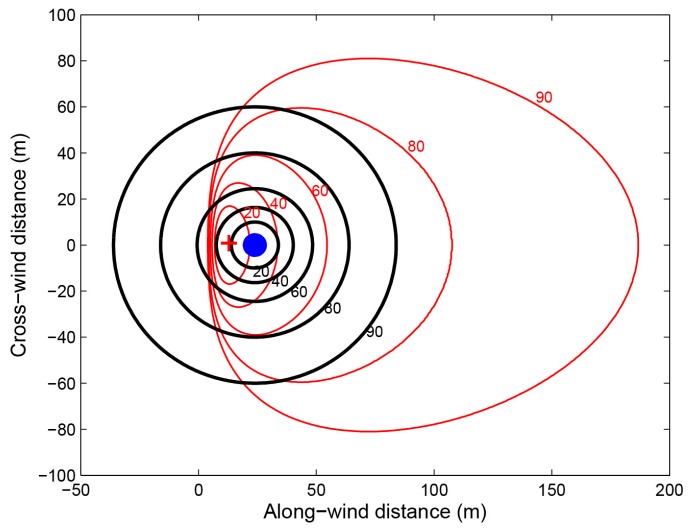
Cumulative footprint contours of the flux (red lines) and hemispherical spectral observations made at nadir (black circles) at the Xiao Tangshan EC site for a height above the target surface of 20 m. The blue circular area represents the total surface source area of the conical spectral observations (FOV of 25°). The center of this circle is the location of the EC tower. The cross marks the location of the flux footprint peak.

As Figure 4 shows, for a typical sensor height above the target surface of 20 m, 90% of the total radiation comes from an FOV lying within 72° (the corresponding footprint radius is 61.55 m), which can cover 75.68% of the source area of the total flux signal under convective conditions. In contrast, the total surface source area of the conical observations (FOV of 25°) is a circle with a radius of 4.43 m, which covers only 1.93% of the flux footprint. Therefore, compared to the conical observation, the hemispherical observation has its advantages in the coordinated measurements of spectra and flux.

### 3.2. Evauation of the Modeled ERTPL Using Simulations

Using the simulated irradiance and transmittance at different VZAs, the modeled ERTPL can be evaluated. It should be noted that the ERTPL model is based on the assumption that the surface is isotropic and that the transmittance is linearly related to the RTPL. However, in practice, these assumptions are not valid. First, for typical vegetation, both the reflectance and the SIF emission varies with the viewing and illumination directions [33]. Secondly, the relationship between the transmittance and RTPL can only be modeled by linear functions for a limited range of the view zenith angle. When the view zenith angle is close to 90°, clearly this relation will become nonlinear (as Figure 4 shows). Therefore, in practice, the ERTPL will not be exactly as shown in Equation (9). 

**Figure 5:**
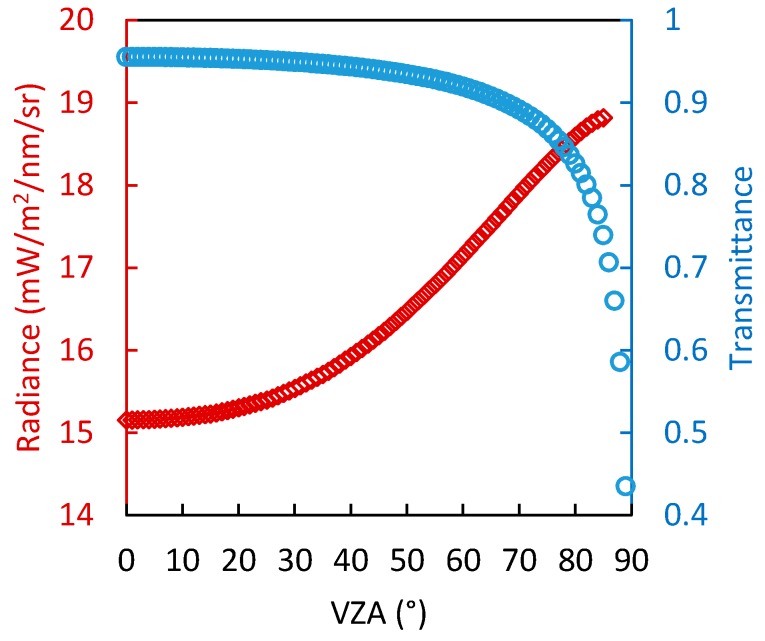
The variation of the up-welling radiance at top of canopy and atmospheric transmittance at 761.1 nm with the view zenith angle simulated by SCOPE and MODTRAN 5 models (Cab = 40 μg/cm^2^, LAI = 4, LIDF is spherical). The spectral resolution is 0.3 nm, the view zenith angle is 0–89°, the relative azimuth angle is 90° and the sensor height above the target surface is 20 m.

Figure 5 shows the variation in the simulated up-welling radiance at top of canopy and atmospheric transmittance of typical vegetation (with an LAI of 4, chlorophyll content of 40 μg/cm^2^, and LIDF of spherical) inside the O_2_-A absorption band (761.1 nm) at different view zenith angles across the solar principal plane simulated by the SCOPE and MODTRAN 5 models. The directional characteristics of up-welling radiance at top of canopy is caused by the bidirectional reflectance effect of the canopy and the directional emission of SIF. The bidirectional reflectance effect of canopy has been widely studied, and has an obvious bowl-edge effect at the far-red band [33,34]. The emission of SIF has also been proved to have similar directional distribution characteristics as reflectance for both observations and simulations [33]. This means that the up-welling radiance at top of canopy will increase as the view zenith angle increases, as shown in Figure 5. In contrast, at the oxygen absorption band, the atmospheric transmittance will fall as the view zenith angle (or RTPL) increases. In other words, the directional characteristics of the up-welling radiance and atmospheric transmittance have opposing influences on the observed upwelling radiance. Therefore, the errors caused by the two assumptions that were made in modeling the ERTPL of the hemispherical measurements will not offset each other to some extent (at least will not accumulate), and the accuracy of the modeled ERTPL value of 2H will be reasonable. 

Using the simulated values of $L_{H}$ and $L_{0}$ for different VZA and RAA, the irradiance observed by a hemispherical measurement system with cosine-corrected foreoptic at a height *H* ($E_{cos}$) above the canopy and at the top of canopy ($E_{0}$) can be calculated by integration using Equations (5) and (7). Hence, the equivalent atmospheric transmittance can be calculated as:

(10) $$\bar{T_{↑}}=\frac{E_{cos}}{E_{0}}=\frac{\int_{0}^{2\pi }\int_{0}^{\frac{\pi }{2}}L_{0}\left(\theta ,\varphi \right)\cdot T_{↑}\left(\theta \right)\cdot \cos\theta \cdot \sin\theta d\theta d\varphi }{\int_{0}^{2\pi }\int_{0}^{\frac{\pi }{2}}L_{0}\left(\theta ,\varphi \right)\cdot \cos\theta \cdot \sin\theta d\theta d\varphi }$$

For bands at atmospheric windows, the influence of atmospheric absorption is very weak. The purpose of this study was to analyze the effect of the atmospheric radiation transfer at the oxygen absorption bands on the SIF retrieval. Therefore, the O_2_-A band (centered at 761.1 nm in the simulated dataset), which is frequently used in SIF retrieval, was selected to evaluate the modeled ERTPL. According to Equation (10), the equivalent atmospheric transmittance of the spectral observations with cosine-corrected foreoptic at a height above the canopy of 20 m is 0.924, and the corresponding ERTPL is 37.7 m (~1.89H), which is close to the modeled ERTPL of 2H.

**Figure 6:**
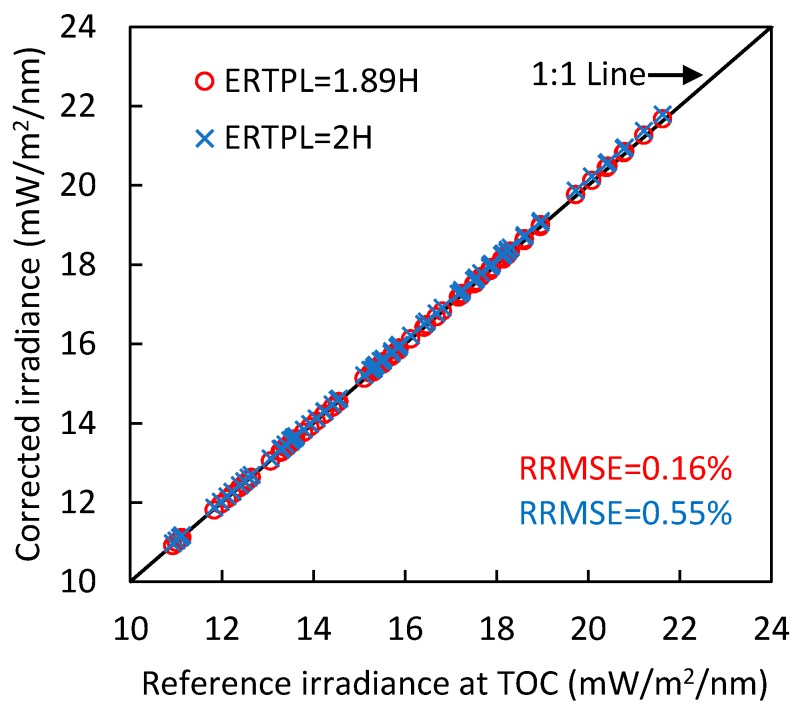
Comparison of irradiance at 761.1 nm corrected using the ERTPL of 1.89H or 2H and the simulated reference irradiance at top of canopy.

The accuracy of atmospheric correction of the hemispherical observation of irradiance at 761.1 nm (within the O_2_-A absorption band) at a height of H above the canopy with the ERTPL of 1.89H or 2H was evaluated by comparing with the simulated reference irradiance at top of canopy, as shown in Figure 6. The scatters of both corrected irradiance with ERTPL of 1.89H and 2H locate close to the 1:1 line, and the RRMSEs are 0.16% and 0.55%, respectively. The difference between the performance of ERTPL of 1.89H and 2H is quite tiny. The results indicate that the ERTPL of 2H modeled in this study is efficient for the atmospheric correction for tower-based hemispherical observation of up-welling irradiance.

### 3.3. Performance of the Atmospheric Correction Using the Modeled ERTPL for SIF Retrieval from the Simulated Dataset

As the fundamental objective of the analysis of the ERTPL of the hemispherical spectral observations was the retrieval of the SIF from tower-based observations, the retrieved SIF from the simulated spectral dataset with 80 different vegetation conditions (as described in Section 2.1) were employed to evaluate the accuracy of the modelled ERTPL. 

The Fraunhofer Line Discrimination (FLD) principle [35] is the mainly used methodology for SIF retrieval at canopy level. Several different FLD-based algorithms have been proposed and applied, such as the standard FLD [35], the 3-bands FLD (3FLD) [36], the improved FLD (iFLD) [37], the principal components analysis based FLD (pFLD) [38], etc. Besides, some spectral fitting methods (SFM) were also proposed and have been proved to be more reliable for SIF retrieval from spectral data with relatively low spectral resolution and signal-to-noise ratio. According to the study by Liu et al. [39], the 3FLD algorithm is robust and simple for SIF retrieval from data with spectral resolution of 0.3 nm, and only three spectral samples are needed. So the 3FLD method was selected for the SIF retrieval from the simulated dataset (with no noise) in this study. Using the 3FLD method, the SIF can be calculated as:

(11) $$SIF_{in}=\frac{\left(I_{left}w_{left}+I_{right}w_{right}\right)L_{in}-I_{in}\left(L_{left}w_{left}+L_{right}w_{right}\right)}{\left(I_{left}w_{left}+I_{right}w_{right}\right)-I_{in}}$$

where *w* is the weight of the band and is inversely proportion to the distance between the left-hand/right-hand band and the inner band; *I* is the downwelling irradiance arriving at the TOC; *L* is the total upwelling radiance at the TOC; and the subscripts “*in*”, “*left*” and “*right*” refer to the bands inside, at the left of and at the right of the absorption band, respectively. 

**Figure 7:**
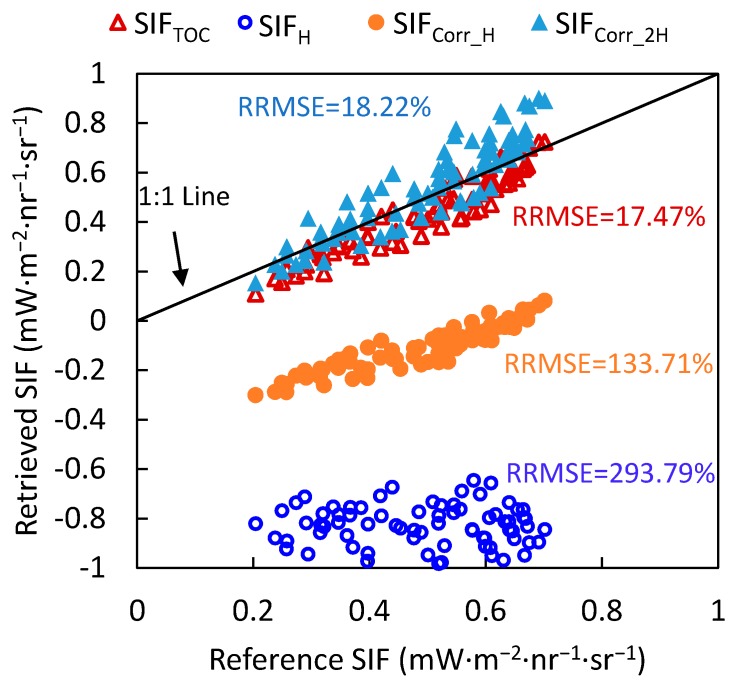
The relation between the modelled reference SIF and the modelled SIF retrieved by the 3FLD method using different atmospheric correction methods. SIF_TOC_ is the SIF retrieved using radiation simulations at the top of the canopy; SIF_H_ is the tower-based SIF retrieved using original spectral simulations at a height of H (20 m) without atmospheric correction; and SIF_corr_H_ and SIF_corr_2H_ are the tower-based SIF retrieved using atmospherically corrected spectra and RTPL values of H and 2H, respectively.

SIF retrieved using radiation simulations at the top of the canopy (SIF_TOC_), tower-based SIF retrieved using original spectral simulations at a height of H (20 m) without atmospheric correction (SIF_H_), and tower-based SIF retrieved using atmospherically corrected spectra and RTPL values of H and 2H (SIF_corr_H_ and SIF_corr_2H_) were compared with the reference values of the simulated SIF, as shown in Figure 7.

As shown in Figure 7, all the SIF_H_ values are negative and located far from the 1:1 line (with a relative root mean square error (RRMSE) of 293.79%), which means that the atmospheric correction was necessary for the tower-based SIF observations although the height of sensor was only 20 m. Compared to the SIF_H_ values, the SIF_corr_H_ values are located much closer to the 1:1 line but the errors are still high (RRMSE = 133.71%). In contrast, the SIF_corr_2H_ values are located close to the 1:1 line (RRMSE = 18.22%) as are the SIF_TOC_ values (RRMSE = 17.47%). It needs to be noted that the ERTPL model proposed in this study overestimates the ERTPL to some extent and, consequently, leads to some overestimation of the SIF. These results indicate that an ERTPL of 2H is suitable for the atmospheric correction of hemispherical observations.

## 4. Discussion

The relationship between SIF and GPP is still not very clear, and there are a lot of uncertainties in both the mechanism and the observations [6]. In this paper, we focused on the match between the footprints of SIF and flux observations. For the tower-based observation of up-welling irradiance of vegetation, there are mainly two different configurations: conical observation and hemispherical observation [8,26]. We compared the footprints of the spectral observations of the two configurations, and modeled the ERTPL of hemispherical observations for atmospheric correction at the oxygen absorption band.

In recent years, more and more automatic tower-based spectral observation systems were established to obtain long-term observations for vegetation in coordination with flux measurements for linking remotely sensed data to ecosystem characteristics [12,13,14,15,16,17]. For those observations, one of the scientific challenges is to determine the most suitable FOV of spectral observations to match with the flux footprint. Balzarolo et al. [11] reviewed the configuration of 55 optical systems at 42 flux tower sites in 2011, and found that 17 out of the 55 systems used hemispherical observations of up-welling irradiance, and the others used the conical observations with FOVs from 5° to 60°.

Porcar-Castell et al. [10] claimed that the hemispherical measurements had great advantage of enabling the sampling of a wider area. According to the results of this paper, 90% of the total radiation comes from an FOV of width 72°, which in turn covered 75.68% of the source area of the flux measurements. So the hemispherical measurements have an obvious advantage to match with the flux footprint. For conical measurements, some alternative ways for better matching with the flux footprint were also proposed. For example, Hilker et al. [40,41] used spectral observations collected over a circular area centered at the flux tower with a rotating system; Gamon et al. [42] introduced a tram system to make spatially representative observations within the flux footprint.

However, there are also a lot of disadvantages for the hemispherical spectral measurements. Firstly, the tower body and its shadow will be in the field of view. Moreover, the influence of them will vary with the illumination geometry [10], which will cause some uncertainties in the observation. For example, if the diameter of the projection of the tower body on the ground is 5 m, and the height of sensor is 20 m, the tower body will cover a FOV of about 14.25° (about 8% of the total FOV of the hemispherical observation). Secondly, the surface of vegetation canopy is not isotropic. Both the reflectance and SIF are directional [33]. For hemispherical observations, radiance from all directions with different weight will be collected. So the directional characteristics of reflectance or SIF should be carefully considered for different cases of application. Thirdly, as the transmittance of cosine-corrected foreoptic is limited (about 25–30% at the far-red band for the Ocean Optics CC-3 cosine corrector), the signal-to-noise ratio of the hemispherical spectral observations will decrease to some extent, which, clearly, could reduce the accuracy of the SIF retrieval.

Another important issue to be considered for the hemispherical observation is the more complex path of radiation transfer. For the conical observation, the FOV is usually very small, and the RTPL can be estimated as the height of the sensor. But for the hemispherical observation, the FOV is 180°, and the ERTPL would be much longer than the sensor’s height. For the tower-based observations for reflectance, wavelengths at atmospheric windows are usually used, so the influence of atmospheric radiation transfer is usually neglected. However, for SIF observation, the atmospheric absorption bands are needed, and the influence of atmosphere is significant [27]. So the ERTPL for tower-based hemispherical observations needs to be modeled. In this paper, according to the theoretical derivation and evaluation with simulated dataset, the ERTPL is modeled as twice of the sensor’s height. The retrieved SIF values from the corrected irradiance using the modeled ERTPL fit well with the simulated reference SIF values. 

It needs to be noted that, the result of the ERTPL for hemispherical observation acquired in this study relies on some assumptions. Firstly, the surface reflectance and SIF emission is assumed to be isotropic whereas, in practice, both reflectance and SIF emission of vegetation has obvious directional characteristics. Moreover, the surface is usually heterogeneous. Secondly, the atmospheric transmittance is assumed to be linearly related to the RTPL, which is only true for a limited range of the view zenith angle. Thirdly, for common setups of tower-based observation, the height of sensor is only tens of meters, and the atmospheric scattering effect is tiny. So the effects of atmospheric scattering were neglected. According to our analysis, the errors caused by the former two assumptions are opposite at the far-red band. Although they cannot totally offset each other, the error will not be accumulated. Finally, this study is totally based on simulation due to the lack of field measurements. Further analysis based on measured dataset should be carried out.

## 5. Conclusions

Using tower-based spectral measurements in coordination with flux measurements is an efficient way of linking SIF to the photosynthesis status. For the observation of up-welling irradiance, both the conical and the hemispherical configurations have their own advantages, but the footprint of hemispherical observation is much wider than the conical observation, and would surely better match with the footprint of flux measurement. However, the effect of atmospheric radiation transfer for hemispherical observation is more complex. In this paper, we developed and evaluated the models of the footprint and ERTPL of hemispherical spectral observations using a simulated dataset and evaluated the performance of the atmospheric correction by using the modeled ERTPL for simulated tower-based SIF retrieval.

First, we developed a method of modeling the footprint of hemispherical spectral observations and found that 90% of the total radiation comes from an FOV of width 72° (the corresponding radius of the footprint is about 3.1 times the sensor’s height above the target surface). For a typical instrument installation height of 20 m above the target surface, and given convective conditions with light winds (0.74 m/s), 90% of the radiation contributing to the hemispherical spectral observations originates from an area that covers 75.68% of the source area of the flux measurements. For conical spectral observations, in contrast, the footprint covers just 1.93% of the flux source area. These results indicate that, when made in conjunction with flux measurements, hemispherical spectral measurements are superior to conical measurements. 

Second, we built a model to estimate the ERTPL of hemispherical spectral observations. Assuming the surface is isotropic and the transmittance is linearly related to the RTPL, the ERTPL of hemispherical spectral observations with cosine-corrected foreoptic can be estimated as being equal to twice of the sensor’s height above the target surface. The modeled ERTPL was evaluated using simulations made by SCOPE and MODTRAN 5. Taking the directional characteristics of up-welling radiance at top of canopy and the non-linear relationship between atmospheric transmittance and RTPL into account, the calculated ERTPL based on the simulations was 1.89H, which is close to the modeled value of 2H. These results indicate that the ERTPL model described in this paper is suitable for making atmospheric corrections to tower-based spectral observations.

Furthermore, the SIF retrieval results based on the simulations also indicate that the modeled ERTPL of 2H is acceptable for use in atmospheric correction. The SIF was retrieved by the 3FLD method using simulated spectra at the top of the canopy, and spectra observed at a height of 20 m without atmospheric correction and with atmospheric corrections using an RTPL of H and 2H. The SIF values retrieved using spectra atmospherically corrected using an RTPL of 2H matched the reference SIF values well—the RRMSE was 18.22%. For the SIF retrieved from spectra that were atmospherically corrected using an RTPL of H and from spectra without atmospheric correction, the RRMSEs were 133.71% and 293.79%, respectively, indicating totally unreliable results. Therefore, the ERTPL model proposed in this paper is helpful for SIF retrieval based on hemispherical spectral measurements. 

In conclusion, considering the match between the footprints of spectral and flux measurements, the hemispherical configuration for the observation of up-welling irradiance has advantage, and the ERTPL for hemispherical observation can be estimated as twice of the sensor’s height above the surface. 

## Figures and Tables

**Table 1:** Main parameters for simulation in SCOPE and MODTRAN 5 models.

Parameter	Description	Value/Range	Unit
Cab	Leaf chlorophyll a + b content	20, 40, 60, 80	μg/cm^2^
LAI	Leaf area index	1, 2, 4, 6	m^2^/m^2^
LIDF	Leaf inclination distribution	Planophile	-
		Erectophile	
		Plagiophile	
		Extremophile	
		Spherical	
SZA	Solar zenith angle	30	degree
VZA	View zenith angle	0–89, in steps of 1	degree
RAA	Relative azimuth angle	0–360, in steps of 10	degree
Height	Surface elevation	20	m
Atmospheric Profile	Atmospheric Profile	Mid-latitude summer	-
AOD550	Aerosol optical depth at 550 nm	0.1	-
Aerosol Model	Aerosol Model	Rural	-
H_2_O	Water vapor column	3	g/cm^2^
O_3_	Ozone column	300	Dobson unit (DU)

**Table 2:** The FFP inputs and their values, as used for modeling the flux footprint at our test site.

Parameter	Description	Value	Unit
zm	Receptor height	20	m
L	Obukhov length	−100	m
σ_v_	Standard deviation of lateral velocity fluctuations	0.45	m/s
u_*_	Friction velocity	0.3	m/s
h	Planetary boundary layer height	2000	m
u_(zm)_	Mean wind velocity at measurement height	0.74	m/s
